# Psychological mechanisms of stress on achievement motivation in college students—the mediating effect of psychological resilience

**DOI:** 10.3389/fpsyg.2026.1796570

**Published:** 2026-04-08

**Authors:** Yushuang Yuan, Yuting Niu, Shusen Liu, Xinqi Li, Wenhui Zhang, Xueyu Zhao, Haoge Bai, Yan Jin, Hao Sun, Yang Meng

**Affiliations:** 1 School of Mental Health, Jining Medical University, Jining, Shandong, China; 2 Clinical Medical College, Jining Medical University, Jining, Shandong, China; 3 School of Pharmacy, Harbin University of Commerce, Harbin, China; 4 College of Medical Imaging and Laboratory, Jining Medical University, Jining, Shandong, China

**Keywords:** achievement motivation, college students, mediation effect, psychological flexibility, stress

## Abstract

**Objective:**

To explore the psychological mechanism of perceived stress on achievement motivation (including motivation to pursue success and avoid failure) in medical college students, and to verify the partial mediating effect of psychological resilience in this relationship.

**Methods:**

A total of 2,691 medical college students in Jining were investigated by random whole cluster sampling, and assessed with Achievement Motivation Inventory (AMS), Chinese Perceived Stress Scale (CPSS) and Connor-Davidson Psychological Resilience Scale (CD-RISC-10). The mediating effect was tested by Model IV of PROCESS plug-in in SPSS 27.0.

**Results:**

(1) Achievement motivation had no significant difference in demographic characteristics; psychological stress had significant differences in grade, gender, major preference and family annual income (*p* < 0.05); psychological resilience had significant differences in all demographic characteristics (*p* < 0.05). (2) Achievement motivation was significantly negatively correlated with psychological stress (*r* = –0.419, *p* < 0.001), and positively correlated with psychological resilience (*r* = 0.492, *p* < 0.001); psychological stress was significantly negatively correlated with psychological resilience (*r* = –0.655, *p* < 0.001). (3) Psychological resilience played a partial mediating role between stress and achievement motivation: the mediating effect accounted for 59.52% of the total effect on motivation to pursue success and 17.24% on motivation to avoid failure.

**Conclusion:**

Perceived stress is directly negatively associated with college students’ achievement motivation, and is also indirectly associated with it via the reduction of psychological resilience. Enhancing psychological resilience may buffer the negative association between stress and achievement motivation.

## 1 Introduction

Achievement motivation refers to the psychological tendency of an individual to not only be willing to do, but also to strive to achieve a higher standard for what he or she considers important or valuable (Wu et al., 2023). Achievement motivation, as an important topic of psychological research, is the intrinsic motivation of students’ academic success, which influences students’ success and failure to some extent (Chen and Chen, 2017). From an individual point of view, achievement motivation, as the most core psychological demand of modern people, is a psychological mechanism to stimulate self-achievement and motivation, and is also a key factor in determining one’s career success or failure. College students’ achievement motivation focuses on the fields of academic pursuit, career planning, and personal growth, which influences college students’ academic performance (Li, 2024), career development, and self-worth realization, and is the intrinsic motivation that pushes college students to pursue success on campus and in the future society (Cole et al., 2004).

In the modern fast-paced society, college students are facing multiple pressures such as ever-increasing academic weight, competitive employment, and expectations from family and society (Loucas et al., 2025). This constant state of stress is not only associated with the mental health level of college students, but may also be closely related to their achievement motivation (Xie, 2024). When the pressure is in a reasonable range, it can motivate college students to clarify their goals, actively invest time and energy to improve themselves, and obtain a sense of achievement in the pursuit of success, which in turn strengthens the achievement motivation (Karaman and Watson, 2017); if the pressure is too high, college students tend to report more feelings of frustration and anxiety, which are associated with lower willingness to pursue success and lower levels of achievement motivation. This relationship between stress and motivation is consistent with the core connotation of the Yerkes-Dodson law (Gorbatkov, 2009).

This study is based on two core theoretical frameworks: the Yerkes-Dodson law, and Self-Determination Theory (SDT) (Ryan and Deci, 2024). The Yerkes-Dodson law explains the inverted U-shaped relationship between stress intensity and behavioral performance, pointing out that excessive stress will reduce individual motivation and task performance. Self-Determination Theory (SDT) points out that intrinsic motivation is the core of individual achievement motivation, and external environmental factors (e.g., excessive stress) affect achievement motivation by influencing the satisfaction of individual autonomy, competence and relatedness needs (Ryan and Deci, 2000). For medical college students, excessive stress is associated with lower satisfaction of their competence needs (e.g., feeling unable to master professional knowledge) and autonomy needs (e.g., learning arranged by the school), which in turn correlates with lower motivation to pursue success and higher motivation to avoid failure.

In this study, the association between stress and achievement motivation is analyzed through two specific paths: the direct negative association between perceived stress and the level of achievement motivation, and the indirect negative association between perceived stress and achievement motivation via the reduction of the individual’s psychological resilience level.

Psychological resilience (Fletcher and Sarkar, 2013) is the ability of an individual to maintain normal psychological function when facing adversity and recover from it (Herrman et al., 2011). Psychological resilience is not a unidimensional and static construct, but a dynamic, process-oriented and multi-component construct involving the interaction of individual and environmental factors (Fletcher and Sarkar, 2013). It includes individual psychological resources (e.g., emotional regulation ability, self-efficacy) and external support resources (e.g., family support, peer support), and its level will change with the change of stress experience and individual growth. In the college student group, those with high psychological resilience can efficiently regulate their state and turn pressure into motivation for academic progress (Masten, 2001); those with low psychological resilienceare easily disturbed by pressure and develop an aversion to learning (García-Vesga, 2013). This shows that psychological resilience exerts a crucial mediating effect in the process of stress influencing college students’ achievement motivation.

Although existing research focuses on the relationship between stress, psychological resilience and achievement motivation (Baker, 2003), there is still a gap in the exploration of the role mechanisms of the three. Based on the above analysis, this study sets three specific research objectives: (1) To examine the distribution characteristics of perceived stress, psychological resilience and achievement motivation among medical college students with different demographic characteristics; (2) To analyze the correlation among perceived stress, psychological resilience and achievement motivation (including two dimensions of motivation to pursue success and avoid failure); (3) To verify the partial mediating effect of psychological resilience in the relationship between perceived stress and achievement motivation, and clarify the proportion of direct and mediating effects.

## 2 Materials and methods

### 2.1 Participants and data collection

Using random whole cluster sampling, we selected freshmen to senior students from 12 faculties of a medical university in Jining City, with college classes as the basic sampling cluster. All subjects signed the informed consent form before filling in the questionnaire, and the survey was conducted in accordance with the ethical requirements of the Declaration of Helsinki.

Inclusion criteria: (1) age range of 18–25 years old (2) full-time undergraduate students; (3) voluntary participation in the study;

Exclusion criteria: (1) Presence of a history of serious psychological or psychiatric illness (2) Recent experience of major negative life events (3) Inability to effectively complete the questionnaire;

This study was conducted using an online questionnaire standardized scale, specifically from 30 May 2025 to 10 June 2025, and the questionnaire contained basic demographic characteristics, achievement motivation scale, psychological stress scale, and psychological resilience scale.

A total of 3,174 questionnaires were returned, resulting in 2,691 valid questionnaires and a validity rate of 84.8%.

#### 2.1.1 Sample characteristics

Table 1 describes the basic demographic characteristics of the 2,691 college students. The majority of the study subjects are students of the class of 2024, who account for 59.1% of all the samples; the gender ratio of the study subjects is relatively balanced, with the proportion of female students (53.3%) slightly higher than that of male students (46.7%); the majority of the study subjects like their own majors (93.6%), and they are mainly non-only-children (70%); the economic conditions of the families are normally distributed and the majority of them have a middle-income family; the annual family income ranges from “less than 10,000 yuan” to “more than 150,000 yuan.” Family economic conditions are normally distributed and middle-income families predominate, with annual family incomes ranging from “less than 10,000 yuan” to “more than 150,000 yuan.” The motivation to pursue success was 39.86 ± 7.49, the motivation to avoid failure was 39.57 ± 8.10, psychological pressure was 30.44 ± 7.51, strength was 18.12 ± 3.07, and resilience was 11.99 ± 2.15.

**TABLE 1:** Distribution of basic demographic characteristics.

Variable	Form	Frequency	Percent	Average value	Standard deviation
Grade Sex Do you enjoy your profession? Rechoose this major Only child Family annual income Monthly living expenses Family structure Father’s level of education Mother’s level of education	Fourth-year university student	17	0.60		
	Third-year university student	641	23.80		
	Second-year university student	443	16.50		
	First-year university student	1590	59.10		
	Male	1258	46.70		
	Female	1433	53.30		
	No	171	6.40		
	Yes	2520	93.60		
	No	479	17.8		
	Yes	2212	82.2		
	No	1884	70.00		
	Yes	807	30.00		
	Less than $10,000	136	5.10		
	$10,000–$30,000	410	15.20		
	$30,000–$50,000	333	12.40		
	$50,000–$80,000	130	4.80		
	$80,000–$100,000	332	12.30		
	$100,000–$120,000	383	14.20		
	$120,000–$150,000	524	19.50		
	More than $150,000	437	16.20		
	Less than $1,000	202	7.5		
	$1,000–$2,000	1231	45.7		
	$2,000–$3,000	1051	39.1		
	More than $3,000	207	7.7		
	Nuclear family	145	5.4		
	Stem family	7	0.3		
	Single-parent family	293	10.9		
	Blended family	11	0.4		
	DINK family	2052	76.3		
	Empty-nest families	114	4.2		
	Grandparent-led households	21	0.8		
	Single-person households	47	1.7		
	United Family	1	0.0		
	Primary school	239	8.9		
	Junior high school	894	33.2		
	Senior high school	560	20.8		
	Junior college/Undergraduate	472	17.5		
	Master’s degree	450	16.7		
	Doctoral degree	76	2.8		
	Primary school	392	14.6		
	Junior high school	902	33.5		
	Senior high school	490	18.2		
	Junior college/Undergraduate	480	17.8		
	Master’s degree	385	14.3		
	Doctoral degree	42	1.6		
Success-seeking motives				39.86	7.49
Avoiding failure motives				39.57	8.10
Stress				30.44	7.51
Strength				18.12	3.07
Resilience				11.99	2.15

### 2.2 Measurements

#### 2.2.1 Achievement Motivation Scale

The Achievement Motivation Scale (AMS) was originally developed by Norwegian psychologists Gjesme and Nygard (1970), its Chinese version was later revised by Ye et al. (1992), and this scale is widely applied to assess individuals’ motivational tendencies in goal pursuit; it comprises two core dimensions, the first is Motivation to Pursue Success (Ms), which includes items 1–15, the second is Motivation to Avoid Failure (Mf), which covers items 16–30; each item is rated on a 4-point Likert scale, the overall achievement motivation score is derived by subtracting the total Motivation to Avoid Failure score from the total Motivation to Pursue Success score, higher scores reflect a greater inclination toward challenging tasks, while low or negative scores indicate higher risk aversion; in this study, the Cronbach’s alpha coefficient for the Motivation to Pursue Success dimension was 0.891, the coefficient for the Motivation to Avoid Failure dimension was 0.916, and the coefficient for the overall scale was 0.782, these values demonstrate robust internal consistency. For more detailed information, refer to Supplementary material 1.

The achievement motivation score was calculated by subtracting the total score of motivation to avoid failure (Mf) from the total score of motivation to pursue success (Ms), which is the classic scoring method of the AMS scale. However, this difference score method has certain psychometric limitations: it loses the independent information of the two dimensions of achievement motivation and may amplify the measurement error of the scale.

#### 2.2.2 Chinese Perceived Stress Scale

The Perceived Psychological Stress Scale (CPSS) was originally developed as the Perceived Stress Scale (PSS) (Cohen et al., 1983), and its Chinese version was later revised by Yang and Huang (2003); this scale is widely applied to evaluate individuals’ perceived levels of psychological stress in daily life. It consists of 14 items, each rated on a 5-point Likert scale with response options ranging from never (1) to always (5), and items 4, 5, 6, 7, 9, 10, and 13 are reverse-scored. The total score ranges from 0 to 56, where scores of 0–18 indicate low stress perception, 19–37 indicate moderate stress perception, and 38–56 indicate high stress perception. In this study, the Cronbach’s alpha coefficient for the scale was 0.660, which is at the lower limit of the acceptable range (0.60–0.70) for psychological scales. The possible reasons are as follows: (1) The research sample is medical college students, whose stress perception characteristics are different from the general population, and some items of the scale are not fully applicable to medical college students; (2) The scale contains 7 reverse-scored items, which may lead to a slight decrease in the internal consistency coefficient. Although the reliability coefficient is marginal, the CPSS scale is a widely used and validated stress measurement tool, and the large sample size of this study also ensures the statistical validity of the measurement results. For the mediation model, the marginal reliability may lead to a slight underestimation of the correlation between stress and other variables, but the significant mediating effect found in the study still indicates the stable relationship among variables, and the conclusion still has reference value. The CPSS scale measures the overall level of perceived stress in daily life, and does not distinguish different subtypes of stress (e.g., academic stress, economic stress, life stress). Therefore, this study cannot clarify which specific type of stress has the most significant impact on the achievement motivation of medical college students. For more detailed information, refer to Supplementary material 2.

#### 2.2.3 Psychological resilience scale short version (10-item Connor-Davidson Resilience Scale, CD-RISC-10).

The 10-item Connor-Davidson Resilience Scale (CD-RISC-10), the short version of the Psychological Resilience Scale, was originally developed by Connor and Davidson (2003), and its Chinese version was later revised by Zhang et al. (2018) and colleagues; this scale is widely applied to assess individuals’ levels of psychological resilience. It consists of 10 items and includes the two dimensions of strength and resilience; each rated on a 5-point Likert scale. The total score ranges from 0 to 40 for this 10-item version, where higher scores reflect greater psychological resilience, representing the ability to recover quickly from adversity, manage stress effectively and maintain a positive mindset, while lower scores indicate susceptibility to stress and a weaker ability to adapt to change. In this study, the Cronbach’s alpha coefficient for the scale was 0.908, demonstrating excellent internal consistency. For more detailed information, refer to Supplementary material 3.

### 2.3 Data analysis

Data were analyzed using SPSS 27.0 software. Prior to formal analysis, strict data screening and cleaning were conducted to ensure data quality, with the following criteria: (1) Excluding questionnaires with a response time of less than 60 s (considered as perfunctory response); (2) Excluding questionnaires with more than 10% of missing items; (3) Excluding questionnaires with consistent answers to all items (e.g., all items rated as 1 or 5). A total of 3174 questionnaires were collected, and 2691 valid questionnaires were retained after screening, with an effective rate of 84.8%. The absolute skewness of all scale scores was ≤ 3 and kurtosis ≤ 10, meeting the criteria of approximate univariate normality for large sample size (all cells *n* > 100). First, descriptive statistics were conducted to analyze the frequencies and percentages of each variable, with scale scores presented as M ± SD. For different demographic subgroups, variance tests were performed, mainly including independent samples *t*-tests and one-way ANOVA. For dichotomous demographic variables (gender, only child or not, favorite major or not), independent samples *t*-test was used for inter-group comparison; for polytomous demographic variables (grade, annual family income), one-way analysis of variance (ANOVA) was used for comparison. The homogeneity of variance test was conducted before ANOVA, and the test results supported the rationality of the analysis method. Pearson correlation analysis was adopted to examine the correlations among stress, achievement motivation and psychological resilience, and the mediation effect was tested using Model IV of the PROCESS plug-in. The statistical significance level was set at α = 0.05 for all tests in this study.

## 3 Results

### 3.1 Normality test

**FIGURE 1:**
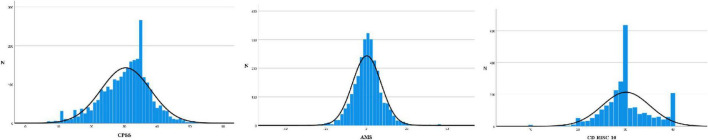
Normal distribution diagram of each scale.

Before constructing the structural equation model, a normality test of the structural model must be conducted to ensure that the probability density distribution of the measured data follows a normal distribution. The normality test includes skewness and kurtosis tests, which are mainly used to judge whether the measured data has good symmetry and peakedness. According to Kline, when the skewness is between ± 3 and the kurtosis is between ± 10, the questionnaire sample data basically follows a normal distribution. Statistical analysis of the distribution characteristics of the sample data was performed using SPSS software, and the results are shown in Table 2. Table 2 presents the skewness and kurtosis values of CPSS, CD-RISC-10, and AMS scales, all of which meet the criteria for normal distribution. The visual distribution of each scale is further illustrated in Figure 1, confirming the normality of the data. The sample data of this questionnaire can be directly used for subsequent analysis.

**TABLE 2:** Normality test.

Variable	Skewness	Std.	Kurtosis	Std.
CPSS	–0.36	0.47	0.494	0.094
CD-RISC-10	0.024	0.47	0.74	0.094
AMS	0.145	0.47	2.645	0.094

### 3.2 Tests of variability

The difference test of demographic variables was conducted to clarify the distribution characteristics of the three core variables in the study sample, which provides a demographic background for the subsequent correlation and mediation analysis. Meanwhile, it explores the influence of demographic factors on perceived stress and psychological resilience, laying a foundation for targeted mental health education for medical college students.

#### 3.2.1 Comparison of AMS scores of respondents with different characteristics

Differential analyses were conducted to examine the differences in achievement motivation across all demographic variables. The results are presented in Table 3, indicating that none of the differences between groups reached statistical significance (*p* > 0.05). The primary purpose of these analyses was to systematically assess the consistency and homogeneity of the sample across subgroups prior to subsequent analyses, thereby verifying whether the sample was representative and suitable for supporting further research. In addition, to ensure the comprehensiveness of the statistical analyses and the integrity of the results presented, all demographic variables were included in the analyses to avoid potential biases associated with selective testing. The non-significant findings collectively demonstrate that the sample did not exhibit systematic differences in the core variable of achievement motivation based on demographic characteristics, indicating good sample consistency and laying a solid foundation for the subsequent analyses.

**TABLE 3:** Comparison of AMS scores of respondents with different characteristics.

Variable	Form	Frequency	Percent	AMS mean score	*t*/*F*	*p*
Grade	Fourth-year university student	17	0.60	–1.24 ± 8.07	0.44	0.726
	Third-year university student	641	23.80	0.58 ± 9.20		
	Second-year university student	443	16.50	0.22 ± 9.72		
	First-year university student	1590	59.10	0.22 ± 8.40		
Sex	Male	1258	46.70	0.45 ± 9.67	0.82	0.411
	Female	1433	53.30	0.16 ± 7.99		
Do you enjoy your profession?	No	171	6.40	–0.66 ± 8.07	–1.47	0.142
	Yes	2520	93.60	0.36 ± 8.86		
Rechoose this major	No	479	17.8	–0.78 ± 0.497	–1.57	0.117
	Yes	2212	82.2			
Only child	No	1884	70.00	0.28 ± 8.68	–0.16	0.872
	Yes	807	30.00	0.34 ± 9.14		
Family annual income	Less than $10,000	136	5.10	0.34 ± 11.03	0.63	0.749
	$10,000–$30,000	410	15.20	0.25 ± 8.24		
	$30,000–$50,000	333	12.40	–0.11 ± 9.38		
	$50,000–$80,000	130	4.80	0.60 ± 9.10		
	$80,000–$100,000	332	12.30	–0.33 ± 8.12		
	$100,000–$120,000	383	14.20	0.74 ± 8.51		
	$120,000–$150,000	524	19.50	0.17 ± 8.01		
	More than $150,000	437	16.20	0.76 ± 9.74		
Monthly living expenses	Less than $1,000	202	7.5	2.47 ± 0.74	0.10	0.960
	$1,000–$2,000	1231	45.7			
	$2,000–$3,000	1051	39.1			
	More than $3,000	207	7.7			
Family structure	Nuclear family	145	5.4	5.61 ± 1.38	0.88	0.531
	Stem family	7	0.3			
	Single-parent family	293	10.9			
	Blended family	11	0.4			
	DINK family	2052	76.3			
	Empty-nest families	114	4.2			
	Grandparent-led households	21	0.8			
	Single-person households	47	1.7			
	United Family	1	0.0			
Father’s level of education	Primary school	239	8.9	3.08 ± 1.33	0.87	0.499
	Junior high school	894	33.2			
	Senior high school	560	20.8			
	Junior college/Undergraduate	472	17.5			
	Master’s degree	450	16.7			
	Doctoral degree	76	2.8			
Mother’s level of education	Primary school	392	14.6	2.88 ± 1.34	1.167	0.323
	Junior high school	902	33.5			
	Senior high school	490	18.2			
	Junior college/Undergraduate	480	17.8			
	Master’s degree	385	14.3			
	Doctoral degree	42	1.6			

#### 3.2.2 Comparison of CPSS scores of respondents with different characteristics

There is a significant difference in psychological pressure in terms of grade (*p* < 0.001), and the psychological pressure of students in grades 2023 and 2024 in the investigated group is higher, while that of students in grades 2021 and 2022 is lower; there is a difference in psychological pressure in terms of different genders (*p* = 0.003), and according to the mean value, it can be seen that the psychological pressure of female students is slightly higher than that of male students. According to the mean value, it can be seen that the psychological pressure of female students is slightly higher than that of male students, and females are more sensitive to pressure; there is a significant difference in psychological pressure on whether they like their majors (*p* < 0.001), and the psychological pressure of students who don’t like their majors is higher in the investigated groups; there is no significant difference in psychological pressure on whether they are only children (*p* = 0.078); there is a significant difference in psychological pressure on the annual income of the family (*p* < 0.001), and the psychological pressure of students with high annual income is higher than that of students with high income in the investigated groups. There is a significant difference in the psychological pressure in terms of annual family income (*p* < 0.001). Please refer to Supplementary material 4 for details

#### 3.2.3 Comparison of CD-RISC-10 scores of respondents with different characteristics

There is a difference in psychological resilience by grade (*p* = 0.003), and the psychological resilience of senior students in the investigated group is higher than that of junior students; there is a significant difference in psychological resilience by gender (*p* < 0.001), and males have higher psychological resilience; there is a significant difference in psychological resilience in terms of whether or not they love their own majors (*p* < 0.001), the psychological resilience of students who love their majors is higher in the surveyed group; there is a significant difference in psychological resilience in whether they are only child (*p* = 0.005), and only child has stronger psychological resilience; there is a significant difference in psychological resilience in the annual income of the family (*p* < 0.001), and the psychological resilience of students with high annual income of the family is higher in the surveyed group. Please refer to Supplementary material 5 for details

### 3.3 Correlation analysis

According to the results of the correlation analysis (Table 4), it can be seen that there is a significant negative correlation between achievement motivation (motivation to pursue success, motivation to avoid failure) and psychological stress (*r* = –0.419, *p* < 0.001) (*r* = 0.514, *p* < 0.001); and there is a significant negative correlation between psychological stress and psychological flexibility (*r* = –0.655, *p* < 0.001); Achievement motivation (motivation to pursue success, motivation to avoid failure) showed a significant positive correlation (*r* = 0.492, *p* < 0.001) with psychological resilience (*r* = 0.430, *p* < 0.001).

**TABLE 4:** Correlation between variables.

Variable	Success-seeking motives	Avoiding failure motives	Stress	Psychological resilience
Success-seeking motives	1	1	1	1
Avoiding failure motives	0.363**			
Stress	–0.419**	–0.541**		
Psychological resilience	0.492**	0.430**	–0.655**	

**p < 0.01.

According to the effect size criteria of Pearson correlation coefficient, the correlation between psychological stress and psychological resilience (*r* = –0.655) is a large effect, and the correlation between psychological stress and motivation to pursue success (*r* = –0.419) is a moderate effect, which indicates that stress is strongly negatively associated with college students’ psychological resilience, and moderately negatively associated with their achievement motivation. This effect size suggests that stress is an important influencing factor of psychological resilience and achievement motivation in medical college students, and has practical significance for mental health education.

### 3.4 Tests for mediating effects

In order to explore the underlying mechanism of the significant negative effect of stress on success-seeking motivation, psychological resilience was further introduced as a mediating variable to be substituted into the structural equation modeling in the study. The test of mediating effect was carried out by using Model IV in the SPSS macro program Process to verify and analyze the mediating role of psychological resilience between psychological stress and achievement motivation (success-seeking motivation, failure-avoidance motivation) according to the method of Bootstrap provided by Hayes.

**FIGURE 2:**
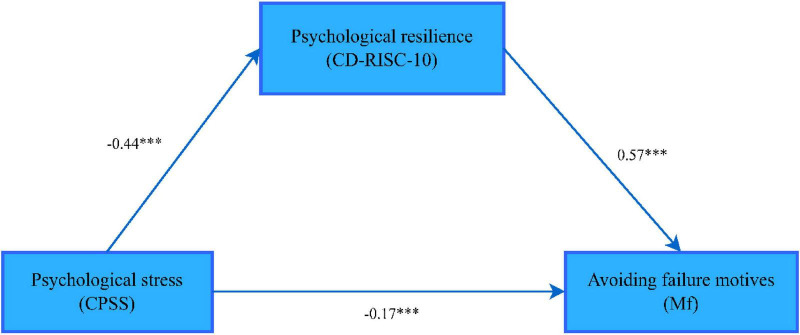
The mediating effect path of psychological resilience between psychological stress and success-seeking motives. ***p < 0.001. The variables are measured by the corresponding scales in parentheses; CPSS, Chinese Perceived Stress Scale, CD-RISC-10, 10-item Connor-Davidson Resilience Scale; AMS, Achievement Motivation Inventory.

The path coefficient of psychological resilience between psychological stress and motivation to pursue success is shown in Figure 2

According to Table 5, the upper and lower limits of the bootstrap 95% confidence intervals for the mediating effect of psychological stress on the motivation to pursue success and psychological resilience do not contain 0, indicating that psychological stress is not only directly associated with the motivation to pursue success, but also indirectly associated with the motivation to pursue success via the variable of psychological resilience. This direct effect (–0.17) and mediating effect (–0.25) account for 40.48 and 59.52% of the total effect (–0.42), respectively.

**TABLE 5:** Breakdown of total, direct and mediating effects.

Effect type	Efficiency value	SE	LLCI	ULCI	Effect size
Aggregate effect	–0.42	0.02	–0.45	–0.38	
Direct effect	–0.17	0.02	–0.21	–0.13	40.48%
Intermediary effect	–0.25	0.02	–0.29	–0.21	59.52%

**FIGURE 3:**
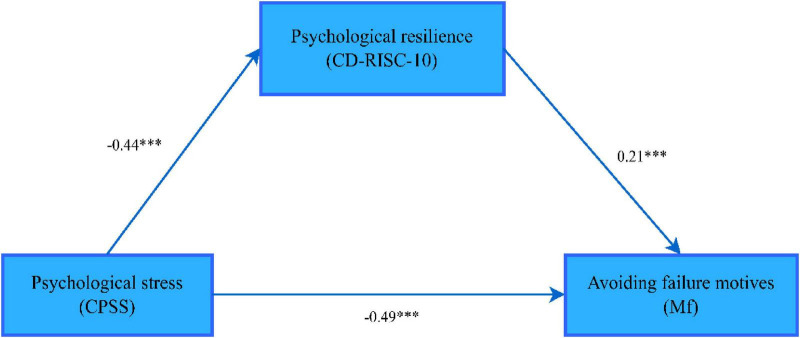
The mediating effect path of psychological resilience between psychological stress and avoiding failure motives. ***p < 0.001. The variables are measured by the corresponding scales in parentheses; CPSS, Chinese Perceived Stress Scale; CD-RISC-10, 10-item Connor-Davidson Resilience Scale; AMS, Achievement Motivation Inventory.

The path coefficient of psychological resilience between psychological stress and motivation to avoid failure is shown in Figure 3

According to Table 6, the upper and lower limits of the bootstrap 95% confidence interval for the mediating effect of psychological stress on the motivation to avoid failure and psychological resilience do not contain 0, indicating that psychological stress is not only directly associated with the motivation to avoid failure, but also indirectly associated with the motivation to avoid failure via the variable of psychological resilience. This direct effect (–0.48) and mediating effect (–0.09) accounted for 82.76 and 17.24% of the total effect (–0.58), respectively. It should be noted that the mediating effect analysis in this study is based on cross-sectional data, which only reveals the mediating relationship among variables at a specific time point and cannot be regarded as evidence of a causal relationship. The causal temporal sequence among perceived stress, psychological resilience and achievement motivation needs to be further verified by longitudinal tracking studies.

**TABLE 6:** Breakdown of total, direct, and mediating effects.

Effect type	Efficiency value	SE	LLCI	ULCI	Effect size
Aggregate effect	–0.58	0.02	–0.62	–0.55	
Direct effect	–0.48	0.02	–0.53	–0.44	82.76%
Intermediary effect	–0.09	0.02	–0.14	–0.05	17.24%

## 4 Discussion and limitations

### 4.1 Discussion

There is a significant negative correlation between achievement motivation and psychological stress, and a significant positive correlation with psychological resilience (Yang and Wang, 2022); there is a significant negative correlation between psychological stress and psychological resilience (Afek et al., 2021). It was found that stress directly and negatively associated with achievement motivation (Ramaprabou and Dash, 2018) on the one hand, and indirectly associated with achievement motivation via lower psychological resilience on the other hand, which suggests that the mediating role of psychological resilience in the model is valid and partially mediated.

#### 4.1.1 Analysis of differences in demographic variables

From the perspective of resilience buffering theory, the differences in psychological resilience among different demographic groups are associated with the differences in individual psychological resources and external environmental support, which in turn correlate with the differences in stress perception. There is no significant difference in achievement motivation (Brunstein and Heckhausen, 2025) by grade, indicating an overall convergence in the pursuit of success and the willingness to avoid failure among students of different grades (Rekha and Praveena, 2015); psychological pressure decreases with increasing grade, which is closely related to the enhancement of experience and skills of students in higher grades, while the grade difference in psychological resilience shows the trajectory of the progressive growth of higher grades in the adaptation to stress. Female college students have higher perceived stress and lower psychological resilience than males, which is consistent with the research results of Graves et al. (2021), Afek et al. (2021), and Souza and Gowda (2016) This gender difference is not a simple social cultural stereotype, but a combination of individual psychological characteristics and social factors: on the one hand, females have higher emotional sensitivity and stronger ability to perceive negative emotions, which is associated with a higher likelihood of reporting stress when facing the same pressure; on the other hand, females have lower active emotional regulation ability than males, and tend to report more difficulty in effectively adjusting their psychological state and mobilizing psychological resources when facing stress, which correlates with lower psychological resilience (Yücel and Torun, 2025), which is because the love of their majors enhances students’ intrinsic motivation and enables them to have more positive experiences in their professional learning, which in turn reduces the perception of stress and improves their psychological adjustment ability; on the contrary, those who are not interested in their majors are prone to resistance in their learning, and the sense of stress accumulates, and it is difficult for them to improve their psychological resilience sufficiently. Only children have higher psychological resilience than non-only children, which is consistent with the research results of Xia et al. (2024). In addition to the sufficient family resource support and attention received by only children, another important alternative explanation is that only children face more independent decision-making and problem-solving scenarios in the growth process (Sheng and Zhu, 2019), which cultivates their better stress adaptation ability and psychological adjustment ability, thus showing higher psychological resilience in the face of college stress. whereas, there is no significant difference between achievement motivation and psychological stress on whether or not they are only children, which suggests that the direct effect of this demographic characteristic on these two variables is weak. Students with high annual family income have lower psychological stress and higher psychological resilience (Haselbach et al., 2025), and the advantage of economic base can provide them with richer learning resources, more relaxed developmental environment, reduce anxiety due to economic pressure, as well as more psychological support in the process of growing up, which can enhance psychological resilience (Yang et al., 2017). Students with high family annual income have lower stress and higher resilience, which is consistent with the research results of Haselbach et al. (2025). The economic advantage can provide students with richer learning resources and a more relaxed developmental environment, reduce anxiety caused by economic pressure, and at the same time provide more psychological support and emotional comfort in the growth process, thus helping students form a higher level of psychological resilience.

#### 4.1.2 The direct effect of stress on college students’ achievement motivation

Consistent with the Yerkes-Dodson law, the study found that excessive perceived stress was significantly negatively associated with college students’ achievement motivation, which may be related to the fact that high perceived stress is associated with lower self-reported psychological resource availability, lower self-efficacy, and less clear goal perception. The results of this study show that there is a significant negative correlation between stress and achievement motivation (pursuit of success motivation, avoidance of failure motivation) (Sharma, 2018). Analyzing from the individual psychological level, individuals with high perceived stress tend to report more anxiety, lower psychological resource availability, lower self-efficacy, and less clear goal perception when facing challenges such as academics and employment, which correlates with lower achievement motivation.; on the contrary, low stress perceivers are able to cope with external challenges in a more positive way, have sufficient psychological resources, strong self-efficacy, and clear goals, and therefore have high achievement motivation (Karaman et al., 2018). This result is also in line with the current situation of college students in modern society, when they are subjected to academic competition, employment pressure and other sustained impacts, if the individual’s perception of stress is high, the achievement motivation will be further weakened, forming a vicious cycle of “high stress—low motivation” (Akram et al., 2011).

#### 4.1.3 The mediating role of psychological resilience

The mediating effect of psychological resilience found in this study reflects the core connotation of dynamic resilience theory (Rutter, 2012): when individuals face continuous stressors (e.g., academic pressure, employment pressure), their resilience level is a dynamic correlational factor—individuals with high resilience tend to report more rapid mobilization of psychological resources for state adjustment, which correlates with a weaker negative association between stress and achievement motivation. Distributional regression tests showed that psychological resilience (Magnano et al., 2016) partially mediated the relationship between both stress and achievement motivation (Trigueros et al., 2019) (success-seeking motivation, failure-avoidance motivation), and hypothesis H3 was tested. Specifically, stress is directly negatively associated with achievement motivation on the one hand, and indirectly negatively associated with achievement motivation via lower psychological resilience on the other hand. Taking the pursuit of success motivation as an example (Grant et al., 2003), higher stress is associated with lower psychological resilience, and lower psychological resilience is further associated with weaker motivation to pursue success, which ultimately correlates with lower levels of motivation to pursue success. The revelation of this mediating mechanism provides a more in-depth perspective for understanding the role of stress on college students’ achievement motivation, and also provides a theoretical basis for colleges and universities to carry out mental health education and academic guidance—if the psychological resilience of college students can be improved through psychological interventions, the negative association between stress and achievement motivation may be buffered to a certain extent (Wang et al., 2025).

### 4.2 Implications

The results of this study provide important empirical basis and practical guidance for the mental health education and achievement motivation cultivation of medical college students. The core practice implication is that colleges and universities should take psychological resilience enhancement as the key point to buffer the negative impact of stress on students’ achievement motivation, and carry out multi-level and targeted psychological intervention strategies combined with the characteristics of medical college students:

1. Curriculum intervention: Set the compulsory course Psychological Resilience Training for Medical Students, which integrates the core technologies of cognitive behavioral therapy (CBT) and mindfulness training. The course focuses on teaching students to identify and correct irrational cognitive beliefs about stress (e.g., “high pressure means failure”), and improve stress regulation ability through mindfulness breathing, body scanning and other methods.

2. Group intervention: Carry out professional-themed psychological group counseling with the theme of “medical professional stress coping” and “medical student career planning,” with 8–10 students in each group. Through peer sharing, role-playing and group discussion, students can learn effective stress coping methods and enhance psychological resilience through peer support.

3. Individual intervention: Establish a psychological early warning system for medical college students, screen students with high stress and low resilience through psychological scales, and provide one-on-one psychological counseling for them. According to the individual stress situation and resilience characteristics, the counselor formulates a personalized intervention plan to solve the specific psychological problems of students.

4. Environmental intervention: Colleges and universities should optimize the teaching management system, reduce the excessive academic competition pressure of medical students, and provide rich academic resources (e.g., professional tutoring, clinical practice opportunities) and employment guidance services. At the same time, build a positive campus psychological atmosphere, and encourage teachers and students to communicate and interact to provide more social support for students.

In addition, colleges should combine the demographic characteristics of students to carry out targeted intervention: for female students, carry out emotional regulation training to improve their stress adaptation ability; for students who do not like their majors, carry out professional cognition education and career planning guidance to enhance their professional identity and intrinsic motivation; for students with low family income, improve the financial aid and scholarship system to reduce their economic pressure.

### 4.3 Limitations

The present study has the following limitations: (1) This study adopted a cross-sectional research design, which only reveals the correlation and mediating relationship among variables at a specific time point, and cannot infer the causal temporal sequence between perceived stress, psychological resilience and achievement motivation. For example, it is impossible to determine whether the decrease of psychological resilience is the result of high stress, or low psychological resilience leads to higher stress perception. Future studies can adopt a longitudinal tracking design to follow up college students from freshmen to graduation, and explore the dynamic causal relationship among the three variables in the process of college growth. (2) It did not differentiate between the specific effects of different types of stressors, such as academic pressure, economic pressure, and so on; (3) The study only focuses on the mediating role of psychological flexibility, without exploring other possible moderating variables (e.g., social support, personality traits, etc.), and the research model can be further expanded in the future to comprehensively analyze the mechanism of stress on achievement motivation. (4) This study measured psychological resilience by CD-RISC-10, which mainly assesses the overall level of resilience and does not distinguish its multi-dimensional components (e.g., individual strength, resilience). The cross-sectional design also cannot reflect the dynamic change process of psychological resilience under the long-term action of stress. Future studies can adopt multi-dimensional resilience scales and longitudinal design to explore the mediating effect of different resilience components on stress and achievement motivation. (5) This study only measured the overall perceived stress of college students and did not explore the differential effects of different stress subtypes (e.g., academic stress, economic stress) on achievement motivation. Medical college students face professional-specific stressors (e.g., clinical practice pressure), and the impact of these specific stressors on achievement motivation needs to be further explored. Future studies can develop a stress scale for medical college students to distinguish stress subtypes and carry out targeted intervention research.

## 5 Conclusion

This study explored the psychological mechanism of perceived stress on achievement motivation in medical college students with psychological resilience as the mediating variable, and obtained three main conclusions: (1) There was no significant difference in achievement motivation among medical college students with different demographic characteristics, while psychological stress and psychological resilience had significant demographic differences: female students, non-major preference students and low family income students had higher stress and lower resilience; senior students and only children had higher psychological resilience. (2) There were significant correlations among perceived stress, psychological resilience and achievement motivation: excessive stress was associated with low achievement motivation and low resilience, while high resilience was associated with high achievement motivation. (3) Psychological resilience played a partial mediating role between perceived stress and achievement motivation: stress is not only directly negatively associated with achievement motivation, but also indirectly negatively associated with it via lower resilience, and the mediating effect was more significant for motivation to pursue success (59.52%) than for motivation to avoid failure (17.24%).

This study enriches the research on the relationship between stress and achievement motivation of college students, and provides a theoretical basis for colleges to carry out mental health education. Enhancing students’ psychological resilience may effectively buffer the negative association between stress and achievement motivation, which is an important way to improve the achievement motivation of medical college students.

## Data Availability

The raw data supporting the conclusions of this article will be made available by the authors, without undue reservation.
