# Supplementary material for: Psychological mechanisms of stress on achievement motivation in college students—the mediating effect of psychological resilience
Source: Front Psychol. 2026 Apr 8;17:1796570. doi: 10.3389/fpsyg.2026.1796570 (PMC13099914; doi:10.3389/fpsyg.2026.1796570)
Supplement: Supplementary file 1 [file Table_1.docx]

**Supplementary Materials**

S1 Chinese version AMS

| 题目 | 完全不符合  （1分） | 有些不符合  （2分） | 基本符合  （3分） | 非常符合  （4分） |
| --- | --- | --- | --- | --- |
| 1、我喜欢新奇的、有困难的任务，甚至不惜冒风险 | 1 | 2 | 3 | 4 |
| 2、我讨厌在完全不能确定会不会失败的情境中工作 | 1 | 2 | 3 | 4 |
| 3、我在完成有困难的任务时，感到快乐 | 1 | 2 | 3 | 4 |
| 4、在结果不明的情况下，我担心失败 | 1 | 2 | 3 | 4 |
| 5、我会被那些能了解自己有多大才智的工作所吸引 | 1 | 2 | 3 | 4 |
| 6、在完成我认为是有困难的任务时，我担心失败 | 1 | 2 | 3 | 4 |
| 7、在结果不明的情况下，我担心失败 | 1 | 2 | 3 | 4 |
| 8、我会被那些能了解自己有多大才智的工作所吸引 | 1 | 2 | 3 | 4 |
| 9、在完成我认为是有困难的任务时，我担心失败 | 1 | 2 | 3 | 4 |
| 10、我喜欢尽了最大努力能完成的工作 | 1 | 2 | 3 | 4 |
| 11、一想到要去做那些新奇的、有困难的工作，我就感到不安 | 1 | 2 | 3 | 4 |
| 12、我喜欢对我没有把握解决的问题坚持不懈地努力 | 1 | 2 | 3 | 4 |
| 13、我不喜欢那些测量我能力的场面 | 1 | 2 | 3 | 4 |
| 14、对于困难的任务，即使没有什么意义，我也很容易 卷进去 | 1 | 2 | 3 | 4 |
| 15、我对那些没有把握能胜任的工作感到忧虑 | 1 | 2 | 3 | 4 |
| 16、面对能测量我能力的机会，我感到是一种鞭策和挑 战 | 1 | 2 | 3 | 4 |
| 17、我不喜欢做我不知道能否完成的事，即使别人不知 道也一样 | 1 | 2 | 3 | 4 |
| 18、我会被有困难的任务所吸引4 | 1 | 2 | 3 | 4 |
| 19、在那些测量我能力的情境中，我感到不安 | 1 | 2 | 3 | 4 |
| 20、对于那些我不能确定是否能成功的工作，最能吸引 我 | 1 | 2 | 3 | 4 |
| 21、对需要有特定机会才能解决的事，我会害怕失败 | 1 | 2 | 3 | 4 |
| 22、给我的任务即使有充裕的时间，我也喜欢立即开始 做 | 1 | 2 | 3 | 4 |
| 23、那些看起来相当困难的事，我做时很担心 | 1 | 2 | 3 | 4 |
| 24、能够测量我能力的机会，对我是有吸引力的 | 1 | 2 | 3 | 4 |
| 25、我不喜欢在不熟悉的环境下工作，即使无人知道也 一样 | 1 | 2 | 3 | 4 |
| 26、面临我没有把握克服的难题时，我会非常兴奋，快 乐 | 1 | 2 | 3 | 4 |
| 27、如果有困难的工作要做，我希望不要分配给我 | 1 | 2 | 3 | 4 |
| 28、如果有事不能立刻理解，我会很快对它产生兴趣 | 1 | 2 | 3 | 4 |
| 29、我不希望做那些要发挥我能力的工作 | 1 | 2 | 3 | 4 |
| 30、对我来说，重要的是做有困难的事，即使无人知道 也无关重要 | 1 | 2 | 3 | 4 |

S2 Chinese version CPSS

| 题目 | 从不(1分) | 偶尔(2分) | 有时(3分) | 时常(4分) | 总是(5分) |
| --- | --- | --- | --- | --- | --- |
| 1.你因为发生一些意外事情，而感到心烦意乱吗？ | 1 | 2 | 3 | 4 | 5 |
| 2.你感到无法控制生活中重要的事情吗？ | 1 | 2 | 3 | 4 | 5 |
| 3.你感到紧张不安和压力大吗？ | 1 | 2 | 3 | 4 | 5 |
| 4.你成功地处理了令人烦恼的生活琐事吗？ | 1 | 2 | 3 | 4 | 5 |
| 5.你感到能够有效地应对生活中发生的重要变化吗？ | 1 | 2 | 3 | 4 | 5 |
| 6.你对于处理自己个人问题的能力感到有信心吗？ | 1 | 2 | 3 | 4 | 5 |
| 7.你感到事情都如你所愿吗？ | 1 | 2 | 3 | 4 | 5 |
| 8.你发现自己无法处理所有你必须做的事情吗？ | 1 | 2 | 3 | 4 | 5 |
| 9.你能够控制自己生活中的恼人事情吗？ | 1 | 2 | 3 | 4 | 5 |
| 10.你感到自己是主宰一切的人吗？ | 1 | 2 | 3 | 4 | 5 |
| 11.你因为许多事情超出你能控制的范围而感到生气？ | 1 | 2 | 3 | 4 | 5 |
| 12.你经常想到有些事情是你必须完成的吗？ | 1 | 2 | 3 | 4 | 5 |
| 13.你能够控制时间运用的方式吗？ | 1 | 2 | 3 | 4 | 5 |
| 14.你感到困难堆积如山，而自己无法克服它们吗？ | 1 | 2 | 3 | 4 | 5 |

S3 Chinese version CD-RISC-10

| 题目 | 从不(0分) | 很少(1分) | 有时(2分) | 经常(3分) | 总是(4分) |
| --- | --- | --- | --- | --- | --- |
| 1、当事情发生变化时，我能够适应 | 0 | 1 | 2 | 3 | 4 |
| 2、无论人生路途中发生任何事情，我都能处理它 | 0 | 1 | 2 | 3 | 4 |
| 3、面临难题时，我试着去看到事情积极的一面 | 0 | 1 | 2 | 3 | 4 |
| 4、经历磨炼会让我更有力量 | 0 | 1 | 2 | 3 | 4 |
| 5、我很容易从疾病、受伤或困难中恢复过来 | 0 | 1 | 2 | 3 | 4 |
| 6、我相信即使遇到障碍我也能够实现我的目标 | 0 | 1 | 2 | 3 | 4 |
| 7、压力之下，我仍然能够集中精神地思考问题 |  | 1 | 2 | 3 | 4 |
| 8、我不会轻易被打败打到 | 0 | 1 | 2 | 3 | 4 |
| 9、在处理生活中的挑战和困难时，我觉得我是个坚强人 | 0 | 1 | 2 | 3 | 4 |
| 10、我能够处理一些不愉快或痛苦的感觉，例如悲伤、生气 | 0 | 1 | 2 | 3 | 4 |

S4 Comparison of CPSS Scores of Respondents with Different Characteristics

| Variable | Form | Frequency | Percent | CPSS mean score | *t*/*F* | *p* |
| --- | --- | --- | --- | --- | --- | --- |
| Grade | Fourth-year university student | 17 | 0.60 | 29.85±7.46 | 12.45 | <0.001 |
|  | Third-year university student | 641 | 23.80 | 29.29±7.42 |  |  |
|  | Second-year university student | 443 | 16.50 | 31.07±7.46 |  |  |
|  | First-year university student | 1590 | 59.10 | 33.12±4.94 |  |  |
| Sex | Male | 1258 | 46.70 | 29.98±7.60 | -2.99 | 0.003 |
|  | Female | 1433 | 53.30 | 30.85±7.41 |  |  |
| Do you enjoy your profession? | No | 171 | 6.40 | 33.78±6.65 | 6.73 | <0.001 |
|  | Yes | 2520 | 93.60 | 30.22±7.51 |  |  |
| Rechoose this major | No | 479 | 17.8 | 30.80±7.32 | 1.14 | 0.255 |
|  | Yes | 2212 | 82.2 | 30.37±7.55 |  |  |
| Only child | No | 1884 | 70.00 | 30.61±7.4 | 1.76 | 0.078 |
|  | Yes | 807 | 30.00 | 30.05±7.74 |  |  |
| Family annual income | Less than $10,000 | 136 | 5.10 | 32.04±8.06 | 7.34 | <0.001 |
|  | $10,000-$30,000 | 410 | 15.20 | 31.94±7.21 |  |  |
|  | $30,000-$50,000 | 333 | 12.40 | 31.00±7.16 |  |  |
|  | $50,000-$80,000 | 130 | 4.80 | 31.32±7.34 |  |  |
|  | $80,000-$100,000 | 332 | 12.30 | 30.81±7.29 |  |  |
|  | $100,000-$120,000 | 383 | 14.20 | 30.38±7.68 |  |  |
|  | $120,000-$150,000 | 524 | 19.50 | 29.55±7.13 |  |  |
|  | More than $150,000 | 437 | 16.20 | 28.73±7.89 |  |  |
| Monthly living expenses | Less than $1,000 | 202 | 7.5 | 29.52±8.17 | 1.59 | 0.19 |
|  | $1,000-$2,000 | 1231 | 45.7 | 30.39±7.57 |  |  |
|  | $2,000-$3,000 | 1051 | 39.1 | 30.56±7.27 |  |  |
|  | More than $3,000 | 207 | 7.7 | 31.07±7.58 |  |  |
| Family structure | Nuclear family | 145 | 5.4 | 31.06±7.32 | 0.71 | 0.685 |
|  | Stem family | 7 | 0.3 | 32.43±3.31 |  |  |
|  | Single-parent family | 293 | 10.9 | 29.74±7.27 |  |  |
|  | Blended family | 11 | 0.4 | 30.36±8.64 |  |  |
|  | DINK family | 2052 | 76.3 | 30.48±7.62 |  |  |
|  | Empty-nest families | 114 | 4.2 | 30.14±6.93 |  |  |
|  | Grandparent-led households | 21 | 0.8 | 32.10±5.52 |  |  |
|  | Single-person households | 47 | 1.7 | 31.02±7.00 |  |  |
|  | United Family | 1 | 0.0 | 33.00±0.00 |  |  |
| Father's level of education | Primary school | 239 | 8.9 | 30.79±7.72 | 2.062 | 0.067 |
|  | Junior high school | 894 | 33.2 | 30.97±7.51 |  |  |
|  | Senior high school | 560 | 20.8 | 30.39±7.00 |  |  |
|  | Junior college/ Undergraduate | 472 | 17.5 | 29.97±7.54 |  |  |
|  | Master’s degree | 450 | 16.7 | 29.79±7.94 |  |  |
|  | Doctoral degree | 76 | 2.8 | 30.33±7.31 |  |  |
| Mother's level of education | Primary school | 392 | 14.6 | 30.66±7.77 | 2.34 | 0.039 |
|  | Junior high school | 902 | 33.5 | 30.79±7.50 |  |  |
|  | Senior high school | 490 | 18.2 | 30.10±6.90 |  |  |
|  | Junior college/ Undergraduate | 480 | 17.8 | 30.69±7.25 |  |  |
|  | Master’s degree | 385 | 14.3 | 39.42±8.00 |  |  |
|  | Doctoral degree | 42 | 1.6 | 31.43±9.37 |  |  |

S5 Comparison of CD-RISC-10 Scores of Respondents with Different Characteristics

| Variable | Form | Frequency | Percent | CD-RISC-10 mean score | *t*/*F* | *p* |
| --- | --- | --- | --- | --- | --- | --- |
| Grade | Fourth-year university student | 17 | 0.60 | 33.12±4.94 | 4.59 | 0.003 |
|  | Third-year university student | 641 | 23.80 | 30.11±5.04 |  |  |
|  | Second-year university student | 443 | 16.50 | 30.67±5.22 |  |  |
|  | First-year university student | 1590 | 59.10 | 29.92±4.95 |  |  |
| Sex | Male | 1258 | 46.70 | 29.98±7.60 | 4.08 | <0.001 |
|  | Female | 1433 | 53.30 | 30.85±7.41 |  |  |
| Do you enjoy your profession? | No | 171 | 6.40 | 28.18±4.89 | -5.23 | <0.001 |
|  | Yes | 2520 | 93.60 | 30.24±5.01 |  |  |
| Rechoose this major | No | 479 | 17.8 | 29.67±5.36 | -2.105 | 0.035 |
|  | Yes | 2212 | 82.2 | 30.21±4.95 |  |  |
| Only child | No | 1884 | 70.00 | 29.93±4.94 | -2.81 | 0.005 |
|  | Yes | 807 | 30.00 | 30.53±5.20 |  |  |
| Family annual income | Less than $10,000 | 136 | 5.10 | 28.96±6.12 | 6.97 | <0.001 |
|  | $10,000-$30,000 | 410 | 15.20 | 29.46±4.53 |  |  |
|  | $30,000-$50,000 | 333 | 12.40 | 29.61±4.78 |  |  |
|  | $50,000-$80,000 | 130 | 4.80 | 29.44±4.92 |  |  |
|  | $80,000-$100,000 | 332 | 12.30 | 29.64±4.82 |  |  |
|  | $100,000-$120,000 | 383 | 14.20 | 30.21±4.88 |  |  |
|  | $120,000-$150,000 | 524 | 19.50 | 30.60±4.90 |  |  |
|  | More than $150,000 | 437 | 16.20 | 31.36±5.44 |  |  |
| Monthly living expenses | Less than $1,000 | 202 | 7.5 | 30.84±4.93 | 1.616 | 0.183 |
|  | $1,000-$2,000 | 1231 | 45.7 | 30.08±5.04 |  |  |
|  | $2,000-$3,000 | 1051 | 39.1 | 30.04±4.99 |  |  |
|  | More than $3,000 | 207 | 7.7 | 29.90 |  |  |
| Family structure | Nuclear family | 145 | 5.4 | 30.32±5.20 | 1.525 | 0.143 |
|  | Stem family | 7 | 0.3 | 28.14±2.55 |  |  |
|  | Single-parent family | 293 | 10.9 | 30.60±5.13 |  |  |
|  | Blended family | 11 | 0.4 | 30.64±5.61 |  |  |
|  | DINK family | 2052 | 76.3 | 30.09±5.01 |  |  |
|  | Empty-nest families | 114 | 4.2 | 29.84±4.78 |  |  |
|  | Grandparent-led households | 21 | 0.8 | 29.24±3.05 |  |  |
|  | Single-person households | 47 | 1.7 | 28.57±5.63 |  |  |
|  | United Family | 1 | 0.0 | 22.00±0.00 |  |  |
| Father's level of education | Primary school | 239 | 8.9 | 30.00±4.61 | 1.10 | 0.356 |
|  | Junior high school | 894 | 33.2 | 29.84±5.16 |  |  |
|  | Senior high school | 560 | 20.8 | 30.19±4.96 |  |  |
|  | Junior college/ Undergraduate | 472 | 17.5 | 30.27±4.94 |  |  |
|  | Master’s degree | 450 | 16.7 | 30.45±5.20 |  |  |
|  | Doctoral degree | 76 | 2.8 | 30.07±4.69 |  |  |
| Mother's level of education | Primary school | 392 | 14.6 | 29.91±5.05 | 0.25 | 0.941 |
|  | Junior high school | 902 | 33.5 | 30.09±4.92 |  |  |
|  | Senior high school | 490 | 18.2 | 30.09±4.82 |  |  |
|  | Junior college/ Undergraduate | 480 | 17.8 | 30.24±5.20 |  |  |
|  | Master’s degree | 385 | 14.3 | 30.23±5.24 |  |  |
|  | Doctoral degree | 42 | 1.6 | 30.14±5.71 |  |  |
